# The impact of COVID-19 on reference services: a national survey of academic health sciences librarians

**DOI:** 10.5195/jmla.2022.1322

**Published:** 2022-01-01

**Authors:** Deborah H. Charbonneau, Emily Vardell

**Affiliations:** 1 dcharbon@wayne.edu, Associate Professor, School of Information Sciences, Wayne State University, Detroit, MI; 2 evardell@emporia.edu, Assistant Professor, School of Library and Information Management, Emporia State University, Emporia, KS

**Keywords:** library services, reference, COVID-19, pandemic, academic libraries

## Abstract

**Objectives:**

The aim of this study was to gain a better understanding of the scope and adaptive nature of reference services provided by academic health sciences librarians over a one-year period (between March 2020 and March 2021) during the COVID-19 pandemic.

**Methods:**

In March 2021, academic health sciences librarians in the United States were invited to participate in an anonymous online survey about their experiences providing reference services during the COVID-19 pandemic. The online survey was developed, pretested, and distributed to various listservs.

**Results:**

A total of 205 academic health sciences librarians and other information professionals with health sciences liaison responsibilities in the US (N=205) responded to the online survey. The scope of reference services provided during the COVID-19 pandemic included email-based reference services (97%), virtual reference (89%), telephone (80%), text-based (33%), and in-person (31%). The most common types of COVID-related reference questions included COVID-19 treatments (53%), safety precautions (46%), vaccines (41%), and prevalence (38%). Additionally, the identification of challenging reference questions and examples of misinformation were provided by respondents.

**Conclusions:**

The results of the survey characterize the evolving nature and scope of academic health sciences reference work during the COVID-19 pandemic. Librarians reported an increase in reference questions during the pandemic and are answering them in creative ways despite barriers (e.g., limited time and reduction in resources). There is an opportunity for librarians to continue to address COVID-related misinformation. Overall, these findings provide useful insight for library practitioners and administrators planning reference services during public health crises.

## INTRODUCTION

The World Health Organization (WHO) declared the alarming number of novel coronavirus cases, commonly known as COVID-19, a global pandemic in March 2020 [1]. During this time, contagion concerns regarding the COVID-19 disease outbreak led to widespread lockdowns across the United States and internationally. The provision of services in many health sciences libraries were disrupted, and librarians worked to adapt to new pandemic information environments.

COVID-19 is caused by the airborne severe acute respiratory syndrome coronavirus 2 (SARS-CoV-2) [2]. The highly contagious nature of COVID-19, as well as the difficulty around understanding how it was transmitted and how to contain it at the beginning of the pandemic, led to many countries adopting social distancing measures and the closures of workplaces, schools, and libraries. Many libraries were completely closed or offered limited in-person services for extended periods of time. With this shift to online work, remote library operations, and more virtual services due to social distancing concerns [3], librarians previously providing services both in person and online were now often working remotely to provide information services for their patrons [4–6].

In addition to this increasingly remote working environment, librarians were also confronted with information needs regarding COVID-19, where authoritative information sources were limited and hard to locate [7]. Particularly at the beginning of the pandemic, but still a concern at the time of this writing, there was an abundance of misinformation concerns about the coronavirus pandemic. The compounding concerns of locating authoritative information about COVID-19, as well as the need to evolve services to fit the unique needs of the time, led to librarian creativity in envisioning redesigned virtual service models [5]. In this rapidly changing information landscape, many libraries reported a transition from providing in-person services to online-only reference and instruction [6,8]. Limited research is available to date documenting how academic health sciences librarians across the US responded to these concerns with a focused investigation on the changing nature of reference services during the COVID-19 pandemic.

To address this gap in the literature, the main objective of this study was to gain a better understanding of the impact of COVID-19 on reference services provided by academic health sciences librarians in the US. In this study, reference work refers to assessing information needs and identifying relevant information and other resources for individuals making the requests [9]. This study examined the scope of reference services, changes to reference work, and the range of reference questions that academic health sciences librarians received amidst the COVID-19 pandemic. The study examined the following research questions:

1. Through which methods did academic health sciences librarians offer reference services (email, virtual, in-person, etc.)?
2. What were the most common changes to reference work due to the pandemic?
3. What were the most common patron groups that academic health sciences librarians provided reference services to?
4. What were the most common types of COVID-related reference questions?
5. What were some of the challenging reference questions or examples of misinformation encountered during reference exchanges?
6. What factors, if any, impacted academic health sciences librarians from performing reference work during the COVID-19 pandemic?

## METHODS

### Study participants and instrument

Of particular interest to the study was the impact of COVID-19 on academic health sciences reference work in the US. Therefore, the study population was academic health sciences librarians and other information professionals with health sciences liaison responsibilities in the US. The ethics committee of Wayne State University approved this study. For the survey, a questionnaire was designed by the authors and developed using Qualtrics software. The online survey instrument was reviewed and pretested by colleagues knowledgeable about the provision of library reference services to help ensure content validity before widely distributing the survey. Survey questions asked respondents about their experiences of providing reference services over a one-year period (March 2020-March 2021) during the COVID-19 pandemic, including the types of reference questions, opinions on how reference work changed during this time, and any factors that may have impacted reference services during the pandemic (Appendix A).

### Data collection and analysis

A nationwide survey was conducted in March 2021. A link to the online survey was distributed to members of the target audience inviting voluntary participation via the MEDLIB-L and Medical Library Association (MLA) chapter listservs [10]. These email listservs presented a viable avenue for recruiting research participants and are open to all MLA members, other interested health sciences librarians, and information specialists as a forum to discuss professional issues [10]. One follow-up email reminder was sent to help increase the number of responses, and data collection concluded after one month at the end of March 2021. Quantitative data obtained through the online survey was exported to Excel for analysis using descriptive statistics. Open-ended narrative responses to the survey questions were also analyzed to identify direct quotes from participants that helped to further explain the quantitative survey results. Next, key findings are presented and summarized.

## RESULTS

A total of 205 academic health sciences librarians and other information professionals with health sciences liaison responsibilities in the US completed the online survey. None of the responses were incomplete; therefore, all responses were included in the analysis (N=205). The majority of the respondents worked in academic health sciences library settings (63%, n=129). This was followed by librarians working in academic library settings with liaison responsibilities to health sciences disciplines (24%, n=49). The remaining respondents worked in other academic settings supporting the health sciences (13%, n=27), such as academic health sciences librarians embedded in departments, working in medical education, informatics centers, or learning resource/technology units on campus.

### Methods of providing reference services

As seen in Table 1, the most common method of providing reference services between March 2020 and March 2021 during the COVID-19 pandemic was by email (97%, n=199). This was followed by virtual/online reference services (89%, n=182) and via telephone (80%, n=164). Only 31% (n=64) of respondents indicated that in-person reference services were offered during this time. Of note, survey respondents reported that their library website was another method of providing information regarding COVID-19 (82%, n=168).

**Table 1:** Methods of providing reference services

Answer	% (n)
Library offered email-based reference services	97% (199)
Library offered virtual/online reference services	89% (182)
Library provided information on its website regarding COVID-19 (e.g., LibGuide, etc.)	82% (168)
Library offered reference services via telephone	80% (164)
Library offered text-based reference services	33% (68)
Library offered in-person reference services	31% (64)

### Changes to reference work

Table 2 illustrates changes to reference work reported by academic health sciences librarians and other information professionals with health sciences liaison responsibilities. Changes to reference work included an increase in virtual/online reference services (84%, n=172) while simultaneously reducing in-person reference services (82%, n=168). With regard to furnishing information resources for COVID-19, 62% of the respondents (n=127) reported identifying and providing general COVID-19 related information while 32% of respondents (n=66) specifically provided information to combat COVID-19 misinformation.

**Table 2:** Changes to reference work

Answer	% (n)
Increased virtual/online reference services	84% (172)
Reduced in-person reference services	82% (168)
Identified/provided general information resources regarding COVID-19	62% (127)
Identified/provided resources to combat misinformation regarding COVID-19	32% (66)
Increased phone reference services	25% (51)
No changes	4% (8)

### Volume of reference questions

**Figure 1:**
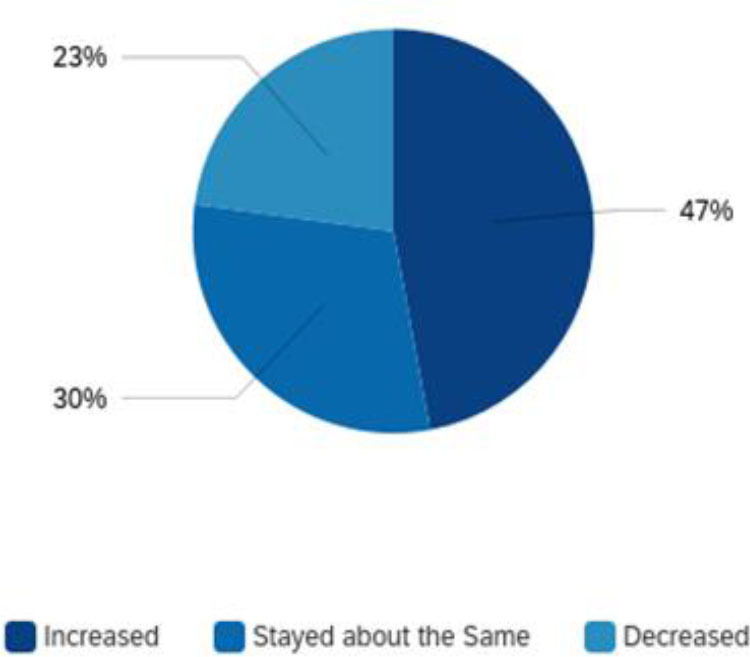
Volume of reference questions

Respondents were asked to indicate the volume of reference questions received during this time either virtually, via phone, email, text, or in person. According to the respondents, 47% (n=96) experienced an increase in reference questions. At the same time, 30% (n=61) reported the amount of reference questions stayed about the same and 23% (n=47) reported a decrease in volume (Figure 1).

### Patron groups served

The most popular audience groups to utilize reference services during this time were faculty (76%, n=156), students (59%, n=121), and researchers (51%, n=105). Interestingly, 17% (n=35) of respondents indicated that reference services were used by community members. Table 3 shows the range of patron groups who used reference services reported by respondents.

**Table 3:** Reference services and patron groups

Answer	% (n)
Faculty	76% (156)
Students	59% (121)
Researchers	51% (105)
Staff	47% (96)
Health care providers	42% (86)
University administrators	22% (45)
General public	17% (35)
Public health department workers	7% (14)

### Types of COVID-related reference questions

Respondents were asked about the types of COVID-related reference questions they had received and were instructed to consider questions asked virtually, via phone, email, text, and in person. The most frequent reference question topic was related to COVID-19 treatments (53%, n=109). This was followed by safety precautions (46%, n=94) and COVID-19 vaccines such as efficacy and side effects (41%, n=84). The ten most common and frequently asked COVID-related reference topics are presented in Table 4.

**Table 4:** Ten most frequent COVID-related reference topics

Answer	% (n)
COVID-19 treatments (e.g., approved drugs, body positioning, protocols, etc.)	53% (109)
Safety precautions (e.g., masks, sanitizer, social distancing, hand washing, etc.)	46% (94)
COVID-19 vaccines (efficacy, side effects)	41% (84)
Prevalence (e.g., number of cases, hospitalizations, deaths, etc.)	38% (78)
COVID-19 symptoms	33% (68)
COVID-19 testing (general, where to get tested, types of tests, etc.)	31% (64)
COVID-19 mandates (local, state, national)	30% (62)
COVID-19 vaccines (general, where to get vaccine)	24% (49)
COVID-19 versus flu	14% (29)
Other (COVID and mental health, stress, and long-term implications)	6% (12)

### Challenging reference questions

Survey participants were asked to reflect on challenging reference questions they received. When it came to reflecting on challenging reference questions, many of the examples centered around the difficulty of locating authoritative information, particularly at the beginning of the pandemic. One respondent shared the question: *“Why do some people get it and some don't even when they were both exposed at the same time?”* Another respondent shared: *“What is the data concerning how SARS-CoV2 causes death? What is the mechanism?”* Another respondent added a concern unique to academic health sciences settings: *“Anatomical dissection during COVID-19. Concerns around COVID+ cadavers and what other medical schools are doing?”* These examples highlight some of the initial concerns surrounding transmission and prognosis and were some of the challenging reference questions.

### Addressing misinformation

Survey participants were asked to share any COVID-related reference questions perceived as dealing with issues of misinformation. Several examples included fact-checking popular news stories and incorrect or incomplete information regarding COVID-19. To help illustrate, one respondent shared the following example: *“The famous question of hydrochloroquinone [sic] effectiveness.”* Another respondent shared: *“Does the vaccine include a microchip for tracking?”* Another reference question was: *“Why do I have to mask and socially distance even after I've had the vaccine, especially since it's been proven that masks don't work?”* An example of a question from a community member was: *“Will COVID-19 disappear in the summer because of higher temperatures?”*

### Factors impacting reference services during COVID-19

Multiple factors were reported by respondents as having an impact on the provision of reference services during the pandemic (Table 5). Librarians were faced with other demands on their time such as additional work due to other assigned duties (59%, n=121) and childcare or their own health concerns during the pandemic (43%, n=88). Additional demands such as the expected turnaround response time for reference questions (31%, n=64) and a reduction in library staff (30%, n=62) were also identified as sources of potential barriers affecting the provision of reference services by health sciences librarians.

**Table 5:** Factors impacting reference services during COVID

Answer	% (n)
Additional work demands on time (e.g., other duties assigned)	59% (121)
Other demands on time (e.g., childcare, personal health concerns, etc.)	43% (88)
Expected turnaround response time	31% (64)
Reduction in library staff	30% (62)
Reduction in library resources	17% (35)
Lack of available information/current evidence	7% (14)

## DISCUSSION

This study resulted in several key findings central to assessing the evolving nature of reference services provided by academic health sciences librarians. First, this study characterized the scope of and changes to reference services over a one-year period during the COVID-19 pandemic. While lockdowns during the global crisis necessitated libraries closing their physical facilities to the public, the adaptive and flexible nature of librarians led to continued provision of essential services and an increase in virtual reference service during pandemic. This finding aligns with previous research noting the shift to virtual library services in academic library settings [11]. As noted by Abubakar [12], “in the midst of the COVID-19 pandemic, which has profoundly affected all forms of physical and social human interactions, the traditional face-to-face reference delivery model has been negatively impacted by lockdowns, isolations, self-quarantines, and physical and social distancing as well.” The present study extends the existing literature to further document the changing and adaptive nature of reference services reported by librarians in academic health sciences environments. While it is not necessarily new for libraries to be transitioning to providing more services remotely, “the scale and speed of the transition to remote work … is unprecedented” [13]. Given that survey respondents reported adapting reference services, increased virtual and phone reference, and continued efforts to maintain continuity of services during the pandemic for various patron groups, this study further demonstrates the role that academic health sciences librarians and information professionals serve in connecting people with the latest information on this emergent topic.

Second, this study found the most frequent reference questions related to COVID-19 were about treatments, safety precautions, vaccines, prevalence, and general symptoms. Libraries can use these findings to meet patron information needs about COVID-19 in a proactive manner. To help address these frequently asked reference questions, libraries can highlight relevant COVID-19 information on library websites, research guides, or other information collections to provide reliable information about the virus. Likewise, the frequent topics could be incorporated into reference training and staff development initiatives to build capacity for current and future scenarios. These findings may also be beneficial to library science faculty educating a future workforce to be responsive to critical information needs stemming from the pandemic.

Third, challenging reference questions and other examples touching on misinformation were highlighted. While there is some overlap in these types of questions, topics ranged from the origin of the virus, exposure risks, transmission modes, and purported treatments, to the efficacy of social distancing measures and wearing masks. These examples offer a snapshot of both health care provider and consumer health concerns during the time of the COVID-19 pandemic and spotlight areas of training for reference librarians. These reference questions present an opportunity to help patrons build their skills at evaluating information sources and verifying COVID-related information in a variety of forums. This may be achieved by locating the best available evidence at the time to combat misinformation regarding COVID-19 as reported by librarians in this study, as well as developing COVID-19 intelligence reports delivering concise, authoritative information updates [14]. Seminars and Q&A sessions as explored in other settings, such as public libraries [15], may also be useful ways to counter the spread of misinformation. Given the risk of misleading or incorrect information, librarians are well positioned to continue to fulfill their mission of supporting patrons by providing access to reliable, trustworthy health information.

Finally, the top factors impacting the ability to perform reference work during the pandemic were revealed, such as time constraints and the reduction in library staff and resources. Due to lockdown and social distancing protocols, many librarians found themselves working remotely either on a full-time basis or in a hybrid mode, and for many this was a new workplace arrangement. With this new working model, librarians were required to balance additional work responsibilities as the situation evolved. As a result, challenges to reference work were made clearer and more visible. Considering the reported barriers to reference services, library administrators can review policies, such as remote and flexible work arrangements, modify operating practices, and determine ways to support library staff in managing workloads and the overall toll of the pandemic. Given the increased use of various technologies to serve library patrons during the pandemic, these findings also suggest there is a need to revisit and potentially restructure reference services. This may involve exploring distributed staffing models (such as tiered reference models), assessing the effectiveness of technologies used for delivering reference services during the global health crisis, and reflecting on the future of reference services [16–17].

While this study illuminated the changing nature of reference services, the study was limited to academic health sciences librarians in the US. The actual size of the population of academic health sciences librarians and other information professionals with health sciences liaison responsibilities in the US is challenging to ascertain; therefore, a response rate could not be determined, and it is unclear to what extent the present findings can be generalized. Nevertheless, this study contributes to understanding how reference services were provided and evolved during this time. Future research should continue to investigate the impact of the COVID-19 pandemic on other library services, such as research support and instruction in academic health sciences libraries. Research in other library settings, such as hospital libraries and public libraries, would also be helpful to grow understanding about how library services are adapting in new pandemic information environments. Since various countries responded to the COVID-19 pandemic with a range of mandates and public health measures, additional research on an international scope is also needed. Future research may consider utilizing additional listservs to expand the potential pool of respondents, such as those hosted by the Association of College & Research Libraries (ACRL), the International Federation of Library Associations and Institutions (IFLA), or others.

## CONCLUSION

In closing, the results of this national survey shed light on the transformative nature of reference services during a significant global event. These findings present an important step in exploring the impact of COVID-19 on academic health sciences reference work and have practical implications for reference training and staff development. A key finding of this study is the most frequently received reference topics that, in turn, can be used to proactively train an information workforce prepared to address COVID-related reference questions and combat misinformation. Another key finding is the identification of factors impacting reference work during the pandemic. Thus, library administrators can use this information to address factors affecting the ability of librarians to perform reference work. Overall, these findings characterize the scope of reference work provided by academic health sciences librarians during the COVID-19 pandemic, document changes to reference work, and provide useful insight for library practitioners and administrators alike planning reference services during public health crises.

## Data Availability

Data associated with this article are available in the Open Science Framework at https://doi.org/10.176Q5/OSF.IO/A7QU9.

## SUPPLEMENTAL FILES

**Appendix A.** Survey questions
