# Supplementary material for: The impact of COVID-19 on reference services: a national survey of academic health sciences librarians
Source: J Med Libr Assoc. 2022 Jan 1;110(1):56–62. doi: 10.5195/jmla.2022.1322 (PMC8830370; doi:10.5195/jmla.2022.1322)
Supplement: Supplementary file 1 — Appendix A. Survey questions [file jmla-110-1-56-s01.docx]

**Appendix A: Survey Questions**

1. In what ways have you provided reference services for the campus community between March 2020 and March 2021 during the COVID-19 pandemic? (Select all that apply.)

- Library offered in-person reference services
- Library offered reference services via telephone
- Library offered virtual/online reference services
- Library offered email-based reference services
- Library offered text-based reference services
- Library provided information on its website regarding COVID-19
- Other:
- N/A

1. In what ways has your reference work changed between March 2020 and March 2021 due to the COVID-19 pandemic? (Select all that apply.)

- Reduced in-person reference services
- Increased virtual/online reference services
- Increased phone reference services
- Provided specialized information for the campus about COVID-19
- Identified/displayed resources to combat mis-information or dis-information regarding COVID-19
- No changes were made to reference services
- Other:
- N/A

1. In your opinion, has the number of ALL reference questions that you received between March 2020 and March 2021 during the COVID-19 pandemic increased, stayed about the same, or decreased?

- Number of reference questions has increased
- Number of reference questions has stayed about the same
- Number of reference questions has decreased

1. Have you received COVID-19 reference questions (between March 2020 and March 2021) from any of the following? (Select all that apply.)

- Students
- Faculty
- Staff
- Researchers
- University administrators
- Health care providers
- Public health department workers
- General public
- Other:
- N/A

1. Please indicate the types of reference question topics that you received related to COVID-19 between March 2020 and March 2021? Please include questions received either virtually, via phone, email, text, and in person. (Select all topics that apply.)

- COVID-19 symptoms
- COVID-19 versus flu
- Prevalence (e.g., number of cases, hospitalizations, deaths, etc.)
- COVID-19 testing (general, where to get tested)
- Safety precautions (e.g., masks, sanitizer, social distancing, hand washing, etc.)
- COVID-19 mandates (e.g., local, state, national)
- COVID-19 treatments (e.g., approved drugs, body positioning, protocols, etc.)
- COVID vaccine (general, where to get vaccine)
- COVID vaccine (efficacy, side effects)
- Other:
- N/A

1. Think back to a challenging reference question that you received related to COVID-19. Please describe the reference question.
2. Think back to a reference question that you received related to misinformation about COVID-19. Please describe the reference question.
3. In your opinion, which of the following factors have impacted your ability to provide reference services between March 2020 and March 2021? (Select all that apply.)

- Expected turnaround response time
- Lack of available information/current evidence
- Reduction in library resources
- Reduction in library staff
- Additional work demands on time (e.g., other duties assigned)
- Other demands on time (e.g., child care, personal health concerns, etc.)
- Other:
- N/A

1. What is your type of library setting? (Select one.)

- Academic health sciences library
- Academic library setting, with liaison responsibilities to health sciences disciplines
- Other
